# Performance of MRI-Based vs Clinical T Staging in Localized Prostate Cancer

**DOI:** 10.1001/jamanetworkopen.2026.23288

**Published:** 2026-07-15

**Authors:** Arthur Peyrottes, Michael Baboudjian, Thibaut Long-Depaquit, Morgan Rouprêt, Thierry Roumeguere, Alexandre Peltier, Guillaume Ploussard, Romain Diamand

**Affiliations:** 1 Department of Urology, Hôpital Saint-Louis, Assistance Publique–Hôpitaux de Paris, Université Paris Cité, Paris, France; 2 Department of Urology, North Hospital, Assistance Publique–Hôpitaux de Marseille, Aix Marseille University, French National Centre for Scientific Research, Interdisciplinary Nanoscience Center of Marseille, ERL INSERM U 1326, European Center for Research in Medical Imaging, Marseille, France; 3 Department of Urology, Saint-Anne Instruction Hospital, Service de Santé des Armées, Toulon, France; 4 Groupe Recherche Clinique 5 Predictive Onco-Urology, Assistance Publique–Hôpitaux de Paris, Pitié-Salpêtrière Hospital, Sorbonne University, Paris, France; 5 Department of Urology, Jules Bordet Institute-Erasme Hospital, Brussels University Hospital, Brussels, Belgium; 6 Department of Urology, La Croix du Sud Hôpital, Quint Fonsegrives, France

## Abstract

**Importance:**

Magnetic resonance imaging (MRI) is increasingly used for local staging in prostate cancer, yet its value relative to digital rectal examination (DRE) staging remains uncertain.

**Objectives:**

To compare the performance of MRI-based vs DRE-based clinical T staging in men undergoing radical prostatectomy (RP) and to assess the outcome of substituting MRI staging in established prognostic classification systems.

**Design, Setting, and Participants:**

This multicenter, retrospective case-control study (iTNM Consortium) was conducted from January 1, 2015, to December 31, 2021, among consecutive men 18 years or older with clinically localized or locally advanced prostate cancer who underwent RP and preoperative multiparametric MRI and systematic biopsies with or without targeted biopsies at 31 referral centers across 6 European countries. Patients receiving prior prostate cancer treatment or missing key variables were excluded. Data analysis was performed from August to October 2025.

**Exposures:**

MRI-derived T stage (iT) vs DRE-derived T stage (cT) incorporated into D’Amico, European Association of Urology, National Comprehensive Cancer Network, and International Staging Collaboration for Prostate Cancer risk groups to generate MRI-adapted classification systems.

**Main Outcomes and Measures:**

Primary outcome was distant metastasis–free survival. Secondary outcomes were biochemical recurrence–free survival and overall survival. Discrimination was assessed using a C index and time-dependent area under the receiver operating characteristic curves.

**Results:**

A total of 4425 men were included (median [IQR] age, 66 [61-70] years; median [IQR] prostate-specific antigen, 7.4 [5.5-10.5] ng/mL). Median (95% CI) follow-up was 52 (51-53) months. MRI-based staging showed slightly higher discriminatory ability than DRE staging for biochemical recurrence–free survival (C index, 0.62 [95% CI, 0.61-0.64] vs 0.59 [95% CI, 0.57-0.61]) and distant metastasis–free survival (0.67 [95% CI, 0.64-0.70] vs 0.65 [95% CI, 0.62-0.68]). However, across all 4 risk classification systems, MRI-based and DRE-based versions demonstrated comparable discrimination with overlapping 95% CIs. Time-dependent area under the receiver operating characteristic curves showed no significant difference during follow-up. Subgroup analyses of patients with cT1 disease and those with PSA levels less than 10 ng/mL yielded similar results.

**Conclusions and Relevance:**

In this case-control study of patients undergoing radical prostatectomy, MRI-based staging demonstrated discrimination similar to traditional DRE-based staging for biochemical recurrence, distant metastasis, and overall survival. These findings suggested that MRI-derived staging could be safely integrated into contemporary risk systems without compromising accuracy.

## Key Points

**Question:**

Does magnetic resonance imaging (MRI)–based T staging provide equivalent discrimination compared with traditional digital rectal examination (DRE) staging for men undergoing radical prostatectomy?

**Findings:**

This case-control study of 4425 men with advanced prostate cancer found that MRI-based staging demonstrated similar discrimination to DRE-based staging for estimating biochemical recurrence–free, distant metastasis–free, and overall survival, with largely overlapping C index 95% CIs across 4 major risk classification systems.

**Meaning:**

This study’s results suggest that MRI-derived staging may replace DRE staging without compromising accuracy, supporting its integration into contemporary risk models in the modern prostate cancer diagnostic pathway.

## Teaser

This case-control study evaluates the performance of magnetic resonance imaging (MRI)–based staging vs traditional digital rectal examination–based staging in men undergoing radical prostatectomy.

## Introduction

Accurate clinical staging of prostate cancer (PCa) is essential for risk stratification, treatment selection, and prognostic counseling. For decades, digital rectal examination (DRE) has served as the cornerstone of local staging, forming the basis of widely used risk classification systems, such as those of D’Amico,^1,2^ the European Association of Urology (EAU),^3^ the National Comprehensive Cancer Network (NCCN),^4^ International Staging Collaboration for Prostate Cancer (STAR-CAP),^5^ and others. However, DRE is inherently limited by its subjectivity, operator dependency, and restricted ability to assess extracapsular extension or seminal vesicle invasion.

Recent evidence has further challenged the role of DRE in contemporary PCa management. Large population-based studies have demonstrated that DRE has limited value as a screening tool^6^ and performs inferiorly to prostate-specific antigen (PSA) testing for cancer detection in systematic evaluation,^7^ intensifying the debate regarding its ongoing role in clinical decision-making. As a result, its relevance in the era of modern imaging is increasingly questioned.^8^

In parallel, the advent of magnetic resonance imaging (MRI) has revolutionized the diagnostic pathway for PCa. Beyond its role in lesion detection and targeted biopsy, MRI provides detailed anatomical information on local tumor extent, enabling noninvasive assessment of extracapsular extension and seminal vesicle invasion. Accordingly, MRI has progressively supplanted DRE as the primary modality for local staging in contemporary practice^9^ and is now recommended in international guidelines before radical treatment.^3,4,10,11^

However, despite its anatomical accuracy, the value of MRI-derived staging remains insufficiently characterized. Although MRI may upstage a substantial proportion of patients compared with DRE,^12^ it remains unclear whether such reclassification translates into improved prediction of clinically relevant outcomes, such as biochemical recurrence, distant metastasis, or overall survival. Prior studies^13,14,15^ addressing this question have been limited by modest sample sizes, single-center design, or lack of long-term follow-up, leaving uncertainty as to whether MRI-based staging truly adds risk estimation information or simply induces stage migration. In this context, we sought to evaluate the performance of MRI-based staging compared with traditional DRE-based staging in men undergoing radical prostatectomy and to assess whether it could safely replace DRE staging in contemporary risk estimation models.

## Methods

### Study Design and Population

This multicenter, retrospective case-control study included consecutive patients with histologically confirmed, clinically localized or locally advanced PCa who underwent radical prostatectomy (RP) between January 1, 2015, and December 31, 2021, across 31 participating institutions in Europe (Belgium, Denmark, France, Germany, Italy, and Switzerland). Each center contributed at least 100 patients to ensure data robustness. The study protocol was approved by the local institutional review board, and data were anonymized before analysis. Overall ethical approval was also obtained from an independent committee from the French Urological Association (Comité d’Éthique de la Recherche en Urologie). Because this study involved retrospective analysis of anonymized data, the requirement for written informed consent was waived by the central institutional review board. This report follows the Strengthening the Reporting of Observational Studies in Epidemiology (STROBE) reporting guideline for case-control studies.

Eligible patients were 18 years or older, had undergone preoperative multiparametric MRI on 1.5T or 3T scanners reported according to Prostate Imaging Reporting and Data System (PI-RADS), version 2.0^16^ or 2.1,^17^ and had undergone systematic biopsies with or without targeted cores (transperineal or transrectal). Patients with negative MRI results (no lesion and PI-RADS scores of 1-2) were included. Exclusion criteria comprised prior therapy for PCa and missing key clinical, radiologic, or pathological variables. RP was performed via open, pure laparoscopic, or robot-assisted approaches, with or without pelvic lymph node dissection, according to local practice.

### Clinical and Imaging Staging

All patients underwent both DRE-based clinical staging and preoperative MRI, allowing direct comparison between cT and iT classifications. Clinical T stage (cT) was determined by DRE in accordance with the 8th edition of *TNM Classification of Malignant Tumours*.^18^ MRI-based extraprostatic extension was defined according to previously published criteria.^19^ Briefly, extracapsular extension was defined by the presence of neurovascular bundle thickening, capsular bulge or irregularity, abutment, or measurable extracapsular disease on T2-weighted images. Seminal vesicle invasion was defined by low signal intensity or abnormal enhancement within or along the seminal vesicle, loss of the angle between the prostate base and seminal vesicle, or direct tumor extension into the seminal vesicle. In the absence of these features, disease was considered organ confined on MRI.

### Risk Classification Systems

Patients were categorized according to 4 established preoperative risk stratification systems: D’Amico,^1^ EAU,^3^ NCCN,^4^ and STAR-CAP.^5^ In addition, a simplified version of the EAU classification (later called EAU simplified) was generated by merging the high-risk and locally advanced categories into a single group to improve statistical stability and facilitate discrimination analyses. For each, a corresponding MRI-based version (iD’Amico, iEAU, iNCCN, and iSTAR-CAP) was constructed by substituting the MRI-derived T stage (iT) for the clinical T stage (cT) using the correspondence proposed by Baboudjian et al^15^ (eFigure 1 in Supplement 1), whereas all other parameters were kept identical. This allowed a direct comparison of performance between the conventional and MRI-adapted systems for the estimation of oncologic outcomes.

### Outcomes and Follow-Up

The primary outcome was distant metastasis–free survival (DMFS), defined as the time from RP to radiologically confirmed distant metastasis (bone or visceral). Secondary outcomes included biochemical recurrence–free survival (BCRFS), defined as the time from surgery to 2 consecutive PSA values greater than 0.2 ng/mL (to convert to micrograms per liter, multiply by 1) after an undetectable postoperative PSA level,^3^ and overall survival (OS), defined as time for RP to death from any cause. Follow-up protocols were based on local practice, typically including serial PSA testing and imaging (computed tomography, bone scintigraphy, or molecular imaging) when clinically indicated. The duration of follow-up was calculated from the date of RP to the date of last contact or death. For DMFS and BCRFS, patients without an event were censored at the last date of imaging or PSA testing, respectively.

### Statistical Analysis

Continuous variables were summarized as medians (IQRs) and categorical variables as numbers (percentages). The median (IQR) follow-up time was calculated using the reverse Kaplan-Meier method,^20^ whereas survival outcomes were estimated using the Kaplan-Meier method. The discriminatory ability of each classification system was assessed using Uno concordance index (C index),^21^ and 95% CIs were estimated via bootstrapping (1000 resamplings). To compare the performance of clinical and MRI-based systems, we computed and contrasted the C indexes for DMFS, BCRFS, and OS. Time-dependent area under the receiver operating characteristic curves (AUCs) were generated to evaluate the evolution of discriminative performance during follow-up. Subgroup analyses were performed in patients with favorable disease: those with negative DRE finding (ie, cT1) or those with a preoperative PSA level less than 10 ng/mL. Statistical significance was considered when 95% CIs did not overlap. All analyses were conducted using R software, version 4.4.2 (R Foundation for Statistical Computing) with the survival, survminer, and timeROC packages. Data analysis was performed from August to October 2025.

## Results

### Patient Characteristics

A total of 4425 men were included. Median (IQR) age at surgery was 66 (61-70) years, and median (IQR) preoperative PSA level was 7.4 (5.5-10.5) ng/mL. At clinical examination, 1610 patients (44.3%) had a positive DRE result, including 131 patients (3.3%) with locally advanced disease. MRI staging yielded higher T categories, with 705 cases (17.0%) classified as iT3a, 130 (3.1%) as iT3b, and 10 (0.2%) as iT4. Reclassification between cT and iT is illustrated in eFigure 2 in Supplement 1. Pathologic stage was pT3a in 1467 patients (34.0%), pT3b in 480 (11.0%), and pT4 in 54 (1.2%). Lymph node invasion and positive surgical margins were observed in 292 individuals (6.6%) and 1265 cases (29.0%), respectively. Complete baseline characteristics are reported in Table 1.

**Table 1.** Baseline Characteristics of the Study Patients

Characteristic	No. (%) of patients^a^ (N = 4425)	Missing data, No.
Age at surgery, median (IQR), y	66 (61-70)	5
Preoperative PSA, median (IQR), ng/mL	7.4 (5.5-10.5)	15
T stage at DRE		
cT1	2176 (55.6)	508
cT2	1610 (41.0)	
cT3	131 (3.3)	
PI-RADS score of index lesion		
No lesion	274 (6.3)	106
3	295 (6.8)	
4	1770 (41.0)	
5	1980 (45.8)	
Maximum lesion diameter of index lesion at MRI, median (IQR), mm	13 (10-18)	756
T stage at MRI		
iT1 (incidental finding)	245 (5.8)	195
iT2	3140 (74.2)	
iT3a	705 (16.7)	
iT3b	130 (3.1)	
iT4	10 (0.2)	
ISUP grade group on biopsy		
1	841 (19.1)	11
2	1895 (42.9)	
3	909 (20.5)	
4	503 (11.4)	
5	246 (5.6)	
Positive cores, median (IQR), %	38 (23-56)	332
Surgical approach		
Open	279 (6.3)	3
Pure laparoscopy	226 (5.1)	
Robot-assisted laparoscopy	3917 (88.6)	
ISUP grade group at final pathology		
1	351 (8.0)	10
2	2124 (48.1)	
3	1288 (29.2)	
4	311 (7.0)	
5	341 (7.7)	
Pathological T stage		
pT2	2333 (53.8)	91
pT3a	1467 (33.8)	
pT3b	480 (11.1)	
pT4	54 (1.2)	
Lymph node invasion at final pathology	292 (6.6)	11
Positive surgical margin	1265 (28.6)	9

Abbreviations: DRE, digital rectal examination; ISUP, International Society of Urological Pathology; MRI, magnetic resonance imaging; PI-RADS, Prostate Imaging Reporting and Data System; PSA, prostate-specific antigen.

SI conversion factor: To convert PSA to µg/L, multiply by 1.

^a^ Unless otherwise indicated.

Median follow-up was 52 months (95% CI, 51-53 months). A total of 3360 patients (77.1%; 95% CI, 75.8%-78.3%) and 1627 patients (36.8%; 95% CI, 35.4%-38.3%) were followed up for at least 3 and 5 years, respectively. At last contact, 1111 patients (25.0%) had experienced biochemical recurrence, and 356 (8.5%) had developed distant metastases. Median time to biochemical recurrence was 106 months (95% CI, 102-113 months), whereas median time to distant metastasis was 118 months (95% CI, 117 months to not reached). Kaplan-Meier curves of BCRFS and DMFS are shown in eFigure 3 in Supplement 1.

### Model Discrimination

Discriminative ability for estimating BCRFS, DMFS, and OS is summarized in Table 2 and Table 3, as well as in eTable 1 in Supplement 1. For BCRFS, the C index of iT was slightly higher than that of cT, reaching 0.62 (95% CI, 0.61-0.64) vs 0.59 (95% CI, 0.57-0.61). When MRI-derived T stages were incorporated into composite risk classification systems, discriminative performance was comparable between MRI-based and classic versions, with overlapping 95% CIs for all comparisons (Table 2). For DMFS, iT yielded a numerically higher C index than DRE-based staging (0.67 [95% CI, 0.64-0.70] vs 0.65 [95% CI, 0.62-0.68]), but the difference was not statistically significant. The MRI-adapted composite systems demonstrated similar discrimination to their standard counterparts (Table 3). For OS, discrimination was modest for all models. MRI-based and DRE-based systems performed similarly, with overlapping CIs for all comparisons (eTable 1 in Supplement 1).

**Table 2.** Comparative Assessment of Model Discrimination for Estimating Biochemical Recurrence–Free Survival After Radical Prostatectomy

Outcome	C index (95% CI)	
	DRE-based models	MRI-based models
T stage (T1 vs T2a vs T2b vs T2c vs T3)	0.59 (0.57-0.61)	0.62 (0.61-0.64)
T stage simplified (T1 vs T2 vs T3)	0.55 (0.54-0.56)	0.58 (0.56-0.59)
D’Amico (low vs intermediate vs high)	0.64 (0.62-0.65)	0.63 (0.61-0.64)
EAU (low vs intermediate-favorable vs intermediate-unfavorable vs high vs locally advanced)	0.66 (0.64-0.68)	0.65 (0.63-0.66)
EAU simplified (low vs intermediate-favorable vs intermediate-unfavorable vs high)	0.66 (0.64-0.67)	0.65 (0.63-0.66)
NCCN (very low vs low vs intermediate-favorable vs intermediate-unfavorable vs high vs very high)	0.61 (0.59-0.62)	0.59 (0.58-0.61)
STAR-CAP (IA vs IB vs IC vs IIA vs IIB vs IIC vs IIIA vs IIIB vs IIIC)	0.68 (0.67-0.70)	0.68 (0.66-0.70)

Abbreviations: DRE, digital rectal examination; EAU, European Association of Urology; MRI, magnetic resonance imaging; NCCN, National Comprehensive Cancer Network; STAR-CAP, International Staging Collaboration for Prostate Cancer.

**Table 3.** Comparative Assessment of Model Discrimination Estimating Distant Metastasis Recurrence–Free Survival After Radical Prostatectomy

Outcome	C index (95% CI)	
	DRE-based models	MRI-based models
T stage (T1 vs T2a vs T2b vs T2c vs T3)	0.65 (0.62-0.68)	0.67 (0.64-0.70)
T stage simplified (T1 vs T2 vs T3)	0.60 (0.57-0.63)	0.61 (0.59-0.64)
D’Amico (low vs intermediate vs high)	0.71 (0.69-0.74)	0.69 (0.67-0.71)
EAU (low vs intermediate-favorable vs intermediate-unfavorable vs high vs locally advanced)	0.74 (0.72-0.76)	0.70 (0.67-0.72)
EAU simplified (low vs intermediate-favorable vs intermediate-unfavorable vs high)	0.72 (0.70-0.74)	0.71 (0.69-0.73)
NCCN (very low vs low vs intermediate-favorable vs intermediate-unfavorable vs high vs very high)	0.66 (0.63-0.69)	0.64 (0.61-0.68)
STAR-CAP (IA vs IB vs IC vs IIA vs IIB vs IIC vs IIIA vs IIIB vs IIIC)	0.78 (0.75-0.81)	0.77 (0.75-0.80)

Abbreviations: DRE, digital rectal examination; EAU, European Association of Urology; MRI, magnetic resonance imaging; NCCN, National Comprehensive Cancer Network; STAR-CAP, International Staging Collaboration for Prostate Cancer.

### Time-Dependent AUC Analyses

**Figure 1.**
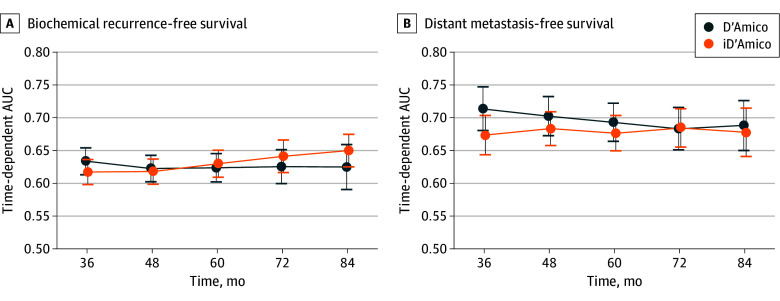
Line Graphs of Time-Dependent Area Under the Curve (AUC) for Prostate Cancer Risk Estimation According to the D’Amico Classification AUCs for the D’Amico classification (digital rectal examination based) and the iD’Amico classification (magnetic resonance imaging based). Error bars indicate 95% CIs.

**Figure 2.**
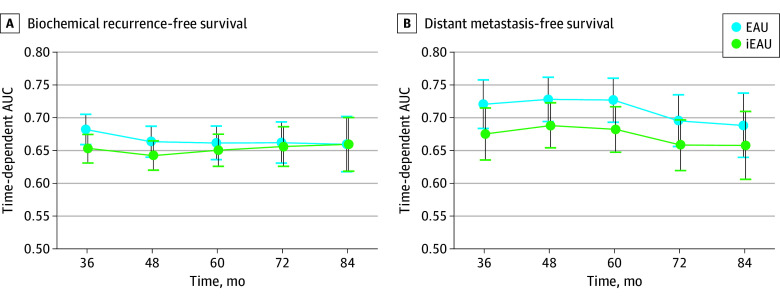
Line Graphs of Time-Dependent Area Under the Curve (AUC) for Prostate Cancer Risk Estimation According to the European Association of Urology (EAU) Classification AUCs for the EAU classification (digital rectal examination based) and the iEAU classification (magnetic resonance imaging based). Error bars indicate 95% CIs.

Time-dependent AUC analyses were performed to assess the evolution of discrimination during follow-up for the D’Amico vs iD’Amico and EAU vs iEAU classification systems. The overall trajectories of the time-dependent AUCs were largely comparable between clinical (DRE-based) and MRI-based models, with overlapping 95% CIs throughout follow-up (Figure 1 and Figure 2).

### Subgroup Analyses

In patients with cT1 disease, discrimination for the risk estimation of both BCRFS and DMFS remained comparable between DRE-based and MRI-based classifications (eTable 2 in Supplement 1). For BCRFS, C indexes were similar across systems, with overlapping 95% CIs (ie, 0.64 [95% CI, 0.62-0.66] for D’Amico vs 0.63 [95% CI, 0.61-0.65] for iD’Amico; 0.68 [95% CI, 0.65-0.70] for EAU vs 0.65 [95% CI, 0.63-0.67] iEAU; 0.68 [95% CI, 0.65-0.70] STAR-CAP vs 0.68 [95% CI, 0.65-0.71] iSTAR-CAP). Comparable findings were observed for DMFS, with no consistent improvement of MRI-based classifications.

In patients with a PSA level less than 10 ng/mL, results were also consistent with those of the overall cohort (eTable 3 in Supplement 1). MRI-based systems achieved similar discrimination to DRE-based models for both BCRFS and DMFS, with nearly identical C indexes and overlapping 95% CIs.

## Discussion

In this large multicenter cohort of 4425 patients treated with RP, MRI-based staging performed similarly to traditional DRE-based staging for estimating BCRFS, DMFS, and OS. Substituting MRI-derived T categories for clinical T stages within major risk classification systems (D’Amico, EAU, NCCN, and STAR-CAP) did not significantly modify discrimination. The time-dependent analyses confirmed comparable performance during follow-up. Subgroup analyses in patients with favorable criteria did not show any difference between clinical and imaging staging.

These findings have several clinical implications. First, they suggest that MRI-based staging, although more anatomically precise, does not provide additional risk estimation information beyond that already captured by PSA level, biopsy International Society of Urological Pathology grade, and other established clinical parameters. This reinforces the robustness of traditional risk systems, which remain valid and reliable in the modern MRI era.^1,3,4,5^ Second, the observation that MRI- and DRE-based systems performed similarly supports the ongoing use of MRI for anatomical delineation and treatment planning (such as nerve-sparing decisions) as well as its integration for local staging in place of DRE in prognostic classifications. Indeed, these results align with previous studies^12,22,23^ suggesting that MRI staging primarily influences treatment planning rather than prognostic prediction. Early work^12,22,23^ demonstrated the superior anatomical accuracy of MRI over DRE for identifying extracapsular extension and seminal vesicle invasion, leading to its integration into routine staging workflows. However, the prognostic value of MRI findings beyond classic clinicobiological factors has remained uncertain. More recent studies^14,24^ have shown that although MRI can upstage a proportion of patients these reclassifications rarely translate into meaningful differences in biochemical or metastasis-free outcomes.^9^ By integrating MRI into established risk systems across a large multinational cohort, our study lends support at scale that this upstaging does not necessarily improve the discriminatory ability of prognostic models but rather reflects a stage migration state.

An additional explanation for the comparable performance between DRE- and MRI-based models may lie in the intrinsic definition of T2 disease across modalities. A cT2 tumor on DRE typically represents a palpable lesion, often posterolateral and clinically significant, and therefore a biologically higher-risk subset of organ-confined disease. Conversely, MRI-defined iT2 encompasses all tumors without radiologic evidence of extracapsular extension or seminal vesicle invasion, including small, anterior, or nonpalpable lesions, resulting in a broader and more prognostically heterogeneous group. This discrepancy likely increases the apparent discriminative value of cT2 relative to iT2, whereas MRI shows its strength in more accurate identification of T3 disease. This stage-specific imbalance may therefore explain why DRE-based models maintain similar performance at the population level despite MRI being anatomically superior.

In this context, we hypothesized that MRI might better stratify patients with apparently low-risk disease (normal DRE or PSA levels <10 ng/mL) by identifying occult extraprostatic extension. However, subgroup analyses did not reveal improved discrimination in these populations. This emphasizes that MRI-visible extraprostatic disease does not necessarily correspond to biologically more aggressive behavior. As such, MRI may refine local staging but remains limited in capturing tumor aggressiveness, which depends on genomic and microenvironmental features not reflected in morphologic imaging.^25^

These findings are particularly relevant given mounting evidence that challenges the clinical utility of DRE in modern PCa care. DRE has recently been shown to provide minimal screening value at a population level^6^ and to perform significantly worse than PSA for cancer detection.^7^ Although historically indispensable for initial evaluation and staging, the role of DRE is therefore being increasingly reconsidered in the MRI era. These findings contribute critical evidence to this paradigm shift, demonstrating that replacing DRE with MRI staging does not compromise oncologic risk discrimination, thereby supporting a transition from historical reliance to evidence-based replacement.

### Strengths and Limitations

The strengths of this study include its large, multicenter design and mid- to long-term follow-up, which enables the capture of distant metastasis (to date, the only validated surrogate in this setting^26^) in the prostate MRI era. The comparison of 4 major classification systems and their MRI-adapted counterparts offers a comprehensive evaluation across different frameworks (through time and space) of clinical risk assessment. However, several limitations should be acknowledged. The retrospective design may entail selection bias and unmeasured confounding. Although MRI scanners, field strengths, and radiologic expertise varied across centers and were not centrally standardized, this heterogeneity arguably reflects community practice rather than introducing bias that would obscure clinically meaningful differences. Similarly, pathological correlation of MRI findings was not uniformly validated across centers. Despite a median follow-up approaching 5 years, longer observation is needed to fully capture the potential long-term oncologic risk estimation contribution of MRI-based staging, particularly for DMFS and OS. Future prospective studies, ideally incorporating information on institutional MRI expertise or radiologist experience, would help clarify whether center-level proficiency influences the performance of MRI-based staging. Finally, the modest discriminative performance observed across all models highlights the intrinsic limitations of current preoperative risk stratification systems, which rely on a limited set of clinical and pathological variables and do not fully capture tumor biological heterogeneity.

## Conclusions

In this case-control study of patients with localized PCa treated with RP, MRI-based staging demonstrated performance similar to traditional DRE-based staging for biochemical recurrence, distant metastasis, and overall survival. These findings suggested that MRI-derived staging could be integrated into contemporary risk classification systems without compromising accuracy. Future prospective studies and incorporation of radiomic or genomic data may further clarify the oncologic risk estimation value of MRI-based staging.

## Notes

Supplement 1. **eFigure 1.** Proposal of a new T classification system based on multiparametric magnetic resonance imaging by Baboudjian et al., European Urology Oncology, 2023 **eFigure 2.** Stage migration between clinical (DRE-based) and MRI-based T classification. **eFigure 3.** Kaplan-Meier curves of A. biochemical recurrence-free survival, and B. distant metastasis-free survival of the whole population. BCR: Biochemical Recurrence **eTable 1.** Comparative assessment of models discrimination for predicting overall survival after radical-prostatectomy **eTable 2.** Comparative assessment of models discrimination for predicting (A) biochemical recurrence-free survival, and (B) distant metastasis-free survival after radical-prostatectomy in patients with negative digital rectal examination (cT1) **eTable 3.** Comparative assessment of models discrimination for predicting (A) biochemical recurrence-free survival, and (B) distant metastasis-free survival after radical-prostatectomy in patients with a PSA<10 ng/ml

Supplement 2. **Nonauthor Collaborators.** Members of the iTNM Consortium

Supplement 3.
Data Sharing Statement
