# Supplementary material for: Performance of MRI-Based vs Clinical T Staging in Localized Prostate Cancer
Source: JAMA Netw Open. 2026 Jul 15;9(7):e2623288. doi: 10.1001/jamanetworkopen.2026.23288 (PMC13373662; doi:10.1001/jamanetworkopen.2026.23288)
Supplement: Supplement 1. — eFigure 1. Proposal of a new T classification system based on multiparametric magnetic resonance imaging by Baboudjian et al., European Urology Oncology, 2023 eFigure 2. Stage migration between clinical (DRE-based) and MRI-based T classification. eFigure 3. Kaplan-Meier curves of A. biochemical recurrence-free survival, and B. distant metastasis-free survival of the whole population. BCR: Biochemical Recurrence eTable 1. Comparative assessment of models discrimination for predicting overall survival after radical-prostatectomy eTable 2. Comparative assessment of models discrimination for predicting (A) biochemical recurrence-free survival, and (B) distant metastasis-free survival after radical-prostatectomy in patients with negative digital rectal examination (cT1) eTable 3. Comparative assessment of models discrimination for predicting (A) biochemical recurrence-free survival, and (B) distant metastasis-free survival after radical-prostatectomy in patients with a PSA<10 ng/ml [file jamanetwopen-e2623288-s001.pdf]

Supplemental Online Content

Peyrottes A, Baboudjian M, Long-Depaquit T, et al. Performance of magnetic resonance imaging–based vs clinical T staging in localized prostate cancer. *JAMA Netw Open*. 2026;9(7):e2623288. doi:10.1001/jamanetworkopen.2026.23288

eFigure 1. Proposal of a new T classification system based on multiparametric magnetic resonance imaging by Baboudjian et al., European Urology Oncology, 2023

eFigure 2. Stage migration between clinical (DRE-based) and MRI-based T classification.

eFigure 3. Kaplan-Meier curves of A. biochemical recurrence-free survival, and B. distant metastasis-free survival of the whole population. BCR: Biochemical Recurrence

eTable 1. Comparative assessment of models discrimination for predicting overall survival after radical-prostatectomy

eTable 2. Comparative assessment of models discrimination for predicting (A) biochemical recurrence-free survival, and (B) distant metastasis-free survival after radical-prostatectomy in patients with negative digital rectal examination (cT1)

eTable 3. Comparative assessment of models discrimination for predicting (A) biochemical recurrence-free survival, and (B) distant metastasis-free survival after radical-prostatectomy in patients with a PSA<10 ng/ml

This supplemental material has been provided by the authors to give readers additional information about their work.

© 2026 Peyrottes A et al. *JAMA Network Open*.

	DRE-based models		MRI-based models	
	**C-index**	**95% CI**	**C-index**	**95% CI**
**T Stage** (T1 *vs* T2a *vs* T2b *vs* T2c *vs* T3)	0.559	0.526-0.605	0.566	0.525-0.611
**T Stage simplified** (T1 *vs* T2 *vs* T3)	0.557	0.523-0.599	0.559	0.509-0.598
**D'Amico** (Low *vs* Intermediate *vs* High)	0.557	0.519-0.597	0.510	0.489-0.552
**EAU** (Low *vs* Intermediate-favorable *vs* Intermediate-unfavorable *vs* High *vs* Locally advanced)	0.579	0.544-0.634	0.538	0.507-0.600
**EAU simplified** (Low *vs* Intermediate-favorable *vs* Intermediate-unfavorable *vs* High)	0.559	0.523-0.606	0.539	0.507-0.589
**NCCN** (Very low *vs* Low *vs* Intermediate-favorable *vs* Intermediate-unfavorable *vs* High *vs* Very high)	0.541	0.515-0.592	0.548	0.516-0.590
**STAR-CAP** (IA *vs* IB *vs* IC *vs* IIA *vs* IIB *vs* IIC *vs* IIIA *vs* IIIB *vs* IIIC)	0.593	0.543-0.638	0.564	0.520-0.615

eTable 1: Models on the left (blue panel) are based on clinical (digital rectal examination) staging, whereas models on the right (red panel) are based on imaging (magnetic resonance imaging) staging.

cT: clinical T staging, iT: imaging T staging, 95% CI: 95% confidence interval, DRE: Digital Rectal Examination, EAU: European Association of Urology, NCCN: National Comprehensive Cancer Network, STARP-CAP: International Staging Collaboration for Prostate Cancer.

© 2026 Peyrottes A et al. *JAMA Network Open*.

A	DRE-based models		MRI-based models	
	C-index	95% CI	C-index	95% CI
**D'Amico**<br>(Low *vs* Intermediate *vs* High)	0.643	0.621-0.663	0.631	0.609-0.652
**EAU**<br>(Low *vs* Intermediate-favorable *vs* Intermediate-unfavorable *vs* High *vs* Locally advanced)	0.675	0.649-0.702	0.651	0.626-0.674
**EAU simplified**<br>(Low *vs* Intermediate-favorable *vs* Intermediate-unfavorable *vs* High)	0.671	0.645-0.694	0.653	0.628-0.674
**NCCN**<br>(Very low *vs* Low *vs* Intermediate-favorable *vs* Intermediate-unfavorable *vs* High *vs* Very high)	0.640	0.615-0.666	0.598	0.569-0.624
**STAR-CAP**<br>(IA *vs* IB *vs* IC *vs* IIA *vs* IIB *vs* IIC *vs* IIIA *vs* IIIB *vs* IIIC)	0.676	0.648-0.704	0.678	0.646-0.705

B	DRE-based models		MRI-based models	
	C-index	95% CI	C-index	95% CI
**D'Amico**<br>(Low *vs* Intermediate *vs* High)	0.730	0.681-0.767	0.696	0.654-0.731
**EAU**<br>(Low *vs* Intermediate-favorable *vs* Intermediate-unfavorable *vs* High *vs* Locally advanced)	0.757	0.717-0.796	0.700	0.657-0.742
**EAU simplified**<br>(Low *vs* Intermediate-favorable *vs* Intermediate-unfavorable *vs* High)	0.748	0.708-0.791	0.710	0.669-0.748
**NCCN**<br>(Very low *vs* Low *vs* Intermediate-favorable *vs* Intermediate-unfavorable *vs* High *vs* Very high)	0.700	0.651-0.748	0.621	0.564-0.681
**STAR-CAP**<br>(IA *vs* IB *vs* IC *vs* IIA *vs* IIB *vs* IIC *vs* IIIA *vs* IIIB *vs* IIIC)	0.768	0.720-0.818	0.761	0.715-0.813

eTable 2: Comparative assessment of models discrimination for predicting (A) biochemical recurrence-free survival, and (B) distant metastasis-free survival after radical-prostatectomy in patients with negative digital rectal examination (cT1). Models on the left (blue panel) are based on clinical (digital rectal examination) staging, whereas models on the right (red panel) are based on imaging (magnetic resonance imaging) staging.

© 2026 Peyrottes A et al. *JAMA Network Open*.

cT: clinical T staging, iT: imaging T staging, 95% CI: 95% confidence interval, DRE: Digital Rectal Examination, EAU: European Association of Urology, NCCN: National Comprehensive Cancer Network, STARP-CAP: International Staging Collaboration for Prostate Cancer.

© 2026 Peyrottes A et al. *JAMA Network Open*.

A	DRE-based models		MRI-based models	
	**C-index**	**95% CI**	**C-index**	**95% CI**
**D'Amico** (Low *vs* Intermediate *vs* High)	0.622	0.602-0.643	0.619	0.597-0.636
**EAU** (Low *vs* Intermediate-favorable *vs* Intermediate-unfavorable *vs* High *vs* Locally advanced)	0.633	0.614-0.658	0.626	0.605-0.647
**EAU simplified** (Low *vs* Intermediate-favorable *vs* Intermediate-unfavorable *vs* High)	0.640	0.618-0.661	0.630	0.608-0.652
**NCCN** (Very low *vs* Low *vs* Intermediate-favorable *vs* Intermediate-unfavorable *vs* High *vs* Very high)	0.622	0.601-0.643	0.590	0.568-0.613
**STAR-CAP** (IA *vs* IB *vs* IC *vs* IIA *vs* IIB *vs* IIC *vs* IIIA *vs* IIIB *vs* IIIC)	0.658	0.633-0.685	0.657	0.632-0.683

B	DRE-based models		MRI-based models	
	**C-index**	**95% CI**	**C-index**	**95% CI**
**D'Amico** (Low *vs* Intermediate *vs* High)	0.701	0.665-0.733	0.684	0.651-0.714
**EAU** (Low *vs* Intermediate-favorable *vs* Intermediate-unfavorable *vs* High *vs* Locally advanced)	0.727	0.686-0.760	0.681	0.644-0.714
**EAU simplified** (Low *vs* Intermediate-favorable *vs* Intermediate-unfavorable *vs* High)	0.714	0.678-0.749	0.707	0.669-0.742
**NCCN** (Very low *vs* Low *vs* Intermediate-favorable *vs* Intermediate-unfavorable *vs* High *vs* Very high)	0.670	0.628-0.713	0.634	0.600-0.678
**STAR-CAP** (IA *vs* IB *vs* IC *vs* IIA *vs* IIB *vs* IIC *vs* IIIA *vs* IIIB *vs* IIIC)	0.762	0.711-0.802	0.754	0.712-0.791

eTable 3: Comparative assessment of models discrimination for predicting (A) biochemical recurrence-free survival, and (B) distant metastasis-free survival after radical-prostatectomy in patients with a PSA < 10 ng/ml. Models on the left (blue panel) are based on clinical (digital rectal examination) staging, whereas models on the right (red panel) are based on imaging (magnetic resonance imaging) staging.

© 2026 Peyrottes A et al. *JAMA Network Open*.

cT: clinical T staging, iT: imaging T staging, 95% CI: 95% confidence interval, DRE: Digital Rectal Examination, EAU: European Association of Urology, NCCN: National Comprehensive Cancer Network, STARP-CAP: International Staging Collaboration for Prostate Cancer.

© 2026 Peyrottes A et al. *JAMA Network Open*.

Stage	Definition
iTx	Primary tumor cannot be assessed
iT0	No tumor
iT1	Incidental diagnosis
iT2	Organ-confined disease
iT2a	PI-RADS ≤3
iT2b	PI-RADS 4
iT2c	PI-RADS 5 without extracapsular extension or seminal vesicle invasion
iT3	Tumor extends through the prostatic capsule
iT3a	Extracapsular extension
iT3b	Seminal vesicle invasion
iT4	Tumor is fixed or invades adjacent structures other than the seminal vesicles: external sphincter, rectum, levator muscles, and/or pelvic wall

PI-RADS = Prostate Imaging-Reporting and Data System.

eFigure 1: Proposal of a new T classification system based on multiparametric magnetic resonance imaging by Baboudjian et al., European Urology Oncology, 2023. PI-RADS: Prostate Imaging Reporting And Data System.

© 2026 Peyrottes A et al. *JAMA Network Open*.

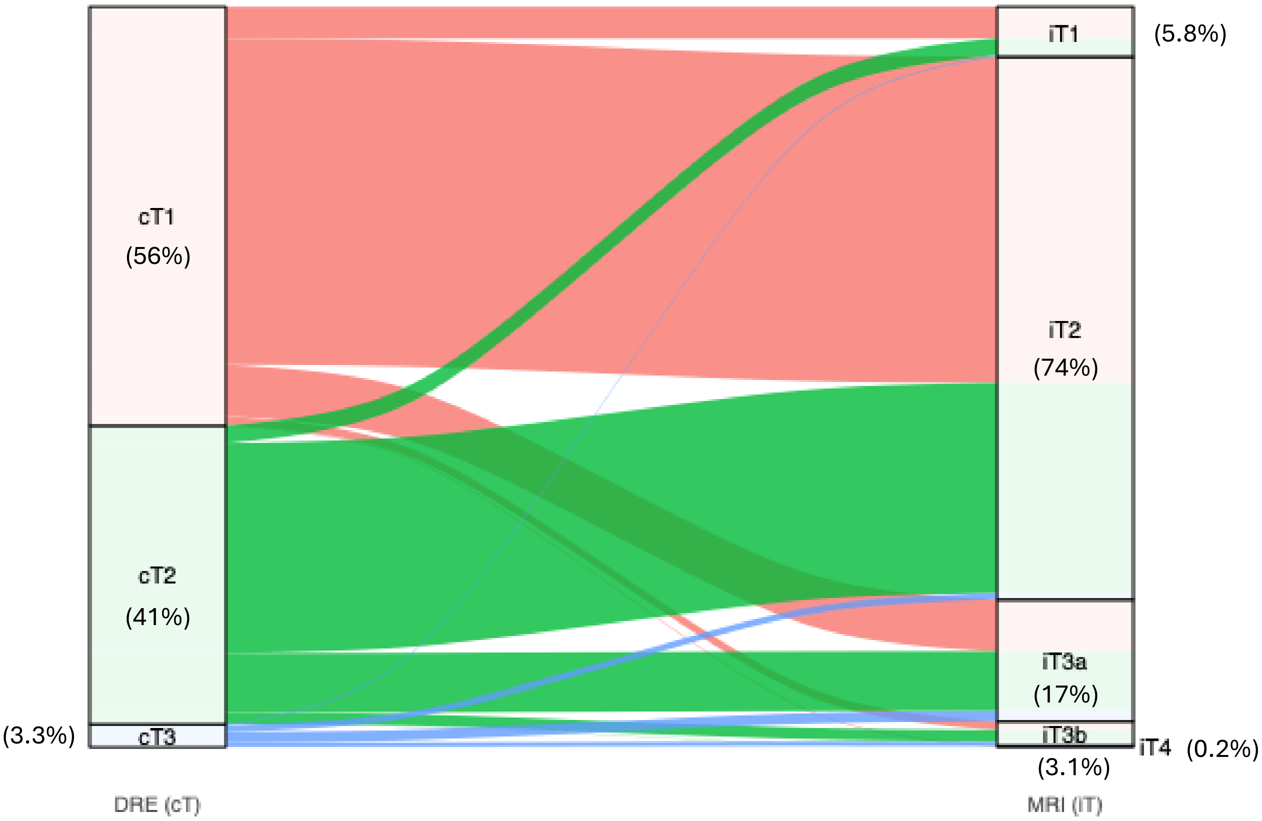

eFigure 2: Stage migration between clinical (DRE-based) and MRI-based T classification.

Flow diagram showing transitions from clinical T stage at digital rectal examination (cT1-3) to MRI-derived T stage (iT1-iT4). The iT2 category aggregates both cT1 and cT2 tumors, reflecting a broader and more heterogeneous organ-confined population, whereas cT2 captures predominantly clinically significant palpable disease. Conversely, MRI increases detection of T3 disease, resulting in frequent upstaging from cT2 to iT3.

© 2026 Peyrottes A et al. *JAMA Network Open*.

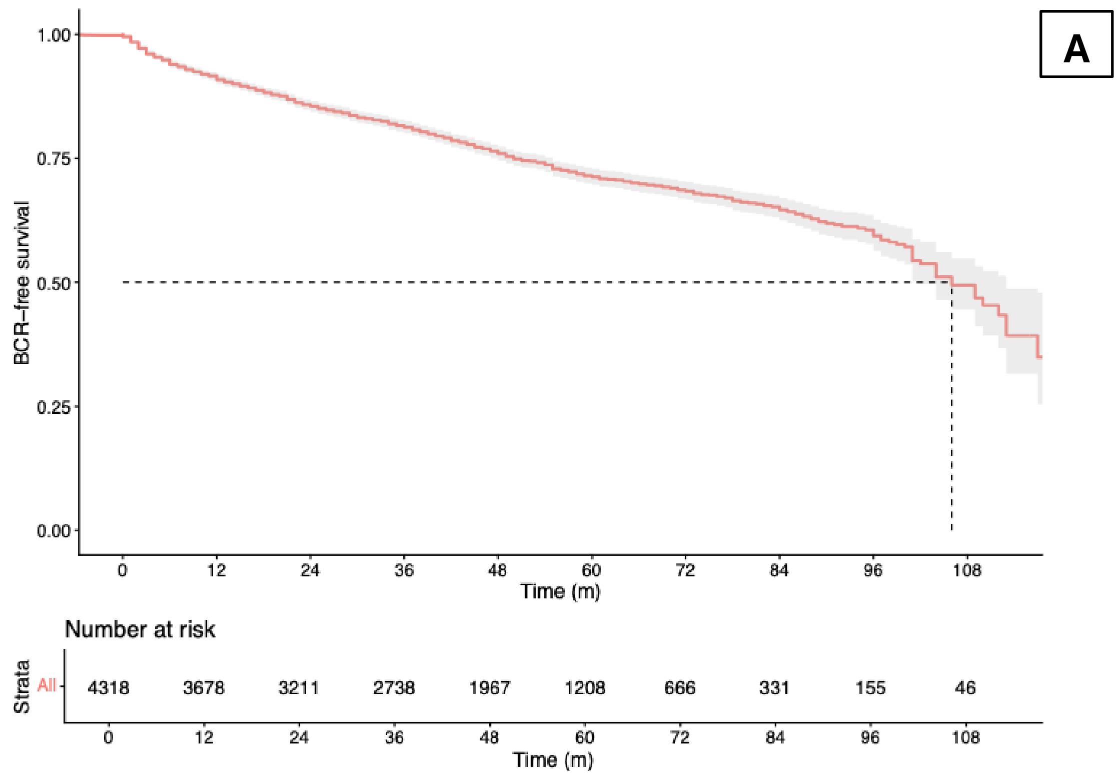

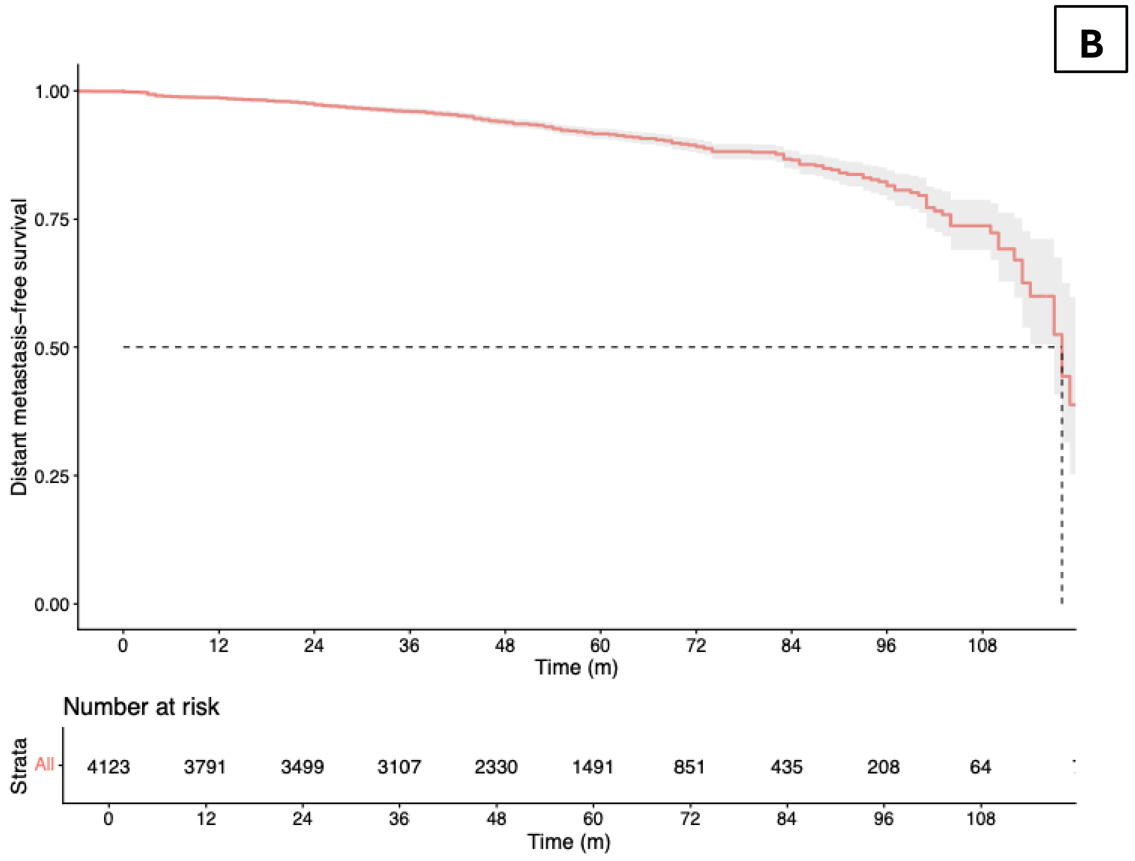

© 2026 Peyrottes A et al. *JAMA Network Open*.

eFigure 3: Kaplan-Meier curves of A. biochemical recurrence-free survival, and B. distant metastasis-free survival of the whole population. BCR: Biochemical Recurrence

Median time to BCR was 106 months (95% CI 102-113), and median time to distant metastasis was 118 months (95% CI 117-not reached).

© 2026 Peyrottes A et al. *JAMA Network Open*.
