# Supplementary material for: Performance of MRI-Based vs Clinical T Staging in Localized Prostate Cancer
Source: JAMA Netw Open. 2026 Jul 15;9(7):e2623288. doi: 10.1001/jamanetworkopen.2026.23288 (PMC13373662; doi:10.1001/jamanetworkopen.2026.23288)
Supplement: Supplement 2. — Nonauthor Collaborators. Members of the iTNM Consortium [file jamanetwopen-e2623288-s002.pdf]

Supplemental Online Content: Nonauthor Collaborators
*First name, last name, and suffix (if applicable) are required and will appear in PubMed.

*Group Name(s): iTNM Consortium							
***First Name and Middle Initial(s)**	***Last Name**	***Suffix (eg, Jr, III)**	Academic Degrees	Institution	Location (city, state/province, country)	Role or Contribution, eg, chair, principal investigator	Group (if more than 1 Group listed in the byline) and/or Subgroup (eg, Steering Committee)
Julien	Anract		M.D., M.Sc.	Department of Urology, Hôpital Cochin, Université Paris-Cité	Paris, France	Principal investigator	
Grégoire	Assenmacher		M.D.	Department of Urology, Clinique St Elisabeth	Brussels, Belgium	Principal investigator	
Michael	Baboudjian		M.D., Ph.D.	Department of Urology, North Hospital, Aix-Marseille University, AP-HM	Marseille, France	Co-Chair	
Francesco	Barletta		M.D.	Division of Oncology/Unit of Urology, Soldera Prostate Cancer Laboratory, Urological Research Institute, IRCCS San Raffaele Scientific Institute	Milan, Italy	Principal investigator	
Eric	Barret		M.D.	Department of Urology, Institut Mutualiste Montsouris	Paris, France	Principal investigator	
Edoardo	Beatrici		M.D.	Department of Urology, AZORG Hospital	Aalst, Belgium	Assistant	
Daniel	Benamran		M.D.	Divison of Urology, Department of Surgery, Geneva University Hospitals, Faculty of Medicine, Geneva University	Geneva, Switzerland	Principal investigator	
Vincent	Benard		M.D.	Divison of Urology, Department of Surgery, Geneva University Hospitals, Faculty of Medicine, Geneva University	Geneva, Switzerland	Assistant	
Nathan	Bergier		M.D.	Cliniques Universitaires Saint-Luc	Brussels, Belgium	Assistant	
Léa	Bollen		M.D.	Department of Urology, Clinique St Elisabeth	Brussels, Belgium	Assistant	
Claudio	Brancelli		M.D.	Department of Urology, Onze Lieve Vrouw Hospital	Aalst, Belgium	Assistant	
Norbert	de Brek		M.D.	Department of Urology, Georges-Pompidou European Hospital, Paris University, U1151 Inserm-INEM, Necker	Paris, France	Assistant	
Laurent	Brureau		M.D., Ph.D.	Department of Urology, Inserm, EHESP, Institut de recherche en santé, environnement et travail (Irset) - UMR_S 1085, CHU de Pointe-à-Pitre, University of Antilles	Guadeloupe, France	Principal investigator	

Supplemental Online Content: Nonauthor Collaborators
*First name, last name, and suffix (if applicable) are required and will appear in PubMed.

***First Name and Middle Initial(s)**	***Last Name**	***Suffix (eg, Jr, III)**	Academic Degrees	Institution	Location (city, state/province, country)	Role or Contribution, eg, chair, principal investigator	Group (if more than 1 Group listed in the byline) and/or Subgroup (eg, Steering Committee)
Marie	Chicaud		M.D., M.Sc.	Department of Urology, Centre Hospitalier Universitaire de Limoges	Limoges, France	Assistant	
Mathieu	Coscarella		M.D.	Department of Urology, Hôpitaux Iris Sud (HIS), Etterbeek-Ixelles Site<br>Department of Urology, Jules Bordet Institute-Erasme Hospital, Brussels University Hospital	Brussels, Belgium	Principal investigator	
Charles	Dariane		M.D., Ph.D.	Department of Urology, Georges-Pompidou European Hospital, Paris University, U1151 - Institut Necker Enfants Malades (INEM), Necker	Paris, France	Principal investigator	
Romain	Diamand		M.D., Ph.D.	Department of Urology, Jules Bordet Institute-Erasme Hospital, Brussels University Hospital	Brussels, Belgium	Co-Chair	
Igor	Duquesne		M.D., M.Sc.	Department of Urology, Hôpitaux Universitaires Henri Mondor, Assistance Publique - Hôpitaux de Paris, Université Paris-Est Créteil+E20:E25	Créteil, France	Principal investigator	
Mariaconsiglia	Ferriero		M.D., Ph.D.	Uro-Oncology program, IRCCS “Regina Elena” National Cancer Institute, Department of Urology	Rome, Italy	Principal investigator	
Gaelle	Fiard		M.D., Ph.D.	University Grenoble Alpes, department of Urology, CNRS, Grenoble INP, TIMC-IMAG, Grenoble-Alpes University Hospital, université Grenoble-Alpes	Grenoble, France	Principal investigator	
Giorgio	Gandaglia		M.D.	Division of Oncology/Unit of Urology, Soldera Prostate Cancer Laboratory, Urological Research Institute, IRCCS San Raffaele Scientific Institute	Milan, Italy	Principal investigator	
Alexander	Giesen		M.D., Ph.D.	Department of Urology, University Hospitals Leuven	Leuven, Belgium	Principal investigator	
Ruben	De Groote		M.D.	Department of Urology, AZORG Hospital	Aalst, Belgium	Principal investigator	
Ruth	Himmelsbach		M.D.	Department of Urology, Faculty of Medicine, University of Freiburg-Medical Centre	Freiburg, Germany	Principal investigator	
Serge	Holz		M.D.	Department of Urology, CHU HELORA	Mons, Belgium	Principal investigator	

Supplemental Online Content: Nonauthor Collaborators
*First name, last name, and suffix (if applicable) are required and will appear in PubMed.

***First Name and Middle Initial(s)**	***Last Name**	***Suffix (eg, Jr, III)**	Academic Degrees	Institution	Location (city, state/province, country)	Role or Contribution, eg, chair, principal investigator	Group (if more than 1 Group listed in the byline) and/or Subgroup (eg, Steering Committee)
Steven	Joniau		M.D., Ph.D.	Department of Urology, University Hospitals Leuven	Leuven, Belgium	Principal investigator	
Marvin	Jourdan		M.D.	Department of Urology, North Hospital, Aix-Marseille University, AP-HM	Marseille, France	Assistant	
Romain	Lefranc		M.D., M.Sc.	Urology department, CHU de Caen	Caen, France	Assistant	
Stéphan	Lévy		M.D., M.Sc.	Department of Urology, CHU-Institut Universitaire du Cancer de Toulouse Oncopole	Toulouse, France	Principal investigator	
Thibaut	Long Depaquit		M.D.	Department of Urology, Hôpital d'Instruction des Armées Saint-Anne	Toulon, France	Principal investigator	
Charles	Mazeaud		M.D., M.Sc.	Department of Urology, IADI-UL-Inserm (U1254), Nancy University Hospital	Nancy, France	Principal investigator	
Benoit	Mesnard		M.D., Ph.D.	Department of Urology and Transplantation Surgery, Nantes University Hospital	Nantes, France	Principal investigator	
Stefano	Moretto		M.D.	Sorbonne Université, GRC 20-Endolase LAB, APHP, Hôpital Tenon, F-75020	Paris, France	Assistant	
Yann	Neuzillet		M.D., Ph.D.	Department of Urology, Hôpital Foch, Université Versailles Saint-Quentin-en-Yvelines	Suresnes, France	Principal investigator	
Myky	Nguyen		M.D.	Department of Urology, Jules Bordet Institute-Erasme Hospital, Brussels University Hospital	Brussels, Belgium	Assistant	
Marco	Oderda		M.D., Ph.D.	Division of Urology, Department of Surgical Sciences, Molinette Hospital, University of Turin	Torino, Italy	Principal investigator	
Jonathan	Olivier		M.D., Ph.D.	Urology Department, Claude Huriez Hospital, CHU	Lille, France	Principal investigator	
Dimitri	Paillusson		M.D.	Department of Urology and Transplantation Surgery, Nantes University Hospital	Nantes, France	Assistant	
Frédéric	Panthier		M.D., Ph.D.	Sorbonne Université, GRC 20-Endolase LAB, APHP, Hôpital Tenon, F-75020	Paris, France	Principal investigator	
Maxime	Pattou		M.D., M.Sc.	Department of Urology, Hôpital Foch, Université Versailles Saint-Quentin-en-Yvelines	Suresnes, France	Assistant	
Arthur	Peyrottes		M.D., M.Sc.	Department of Urology, Hôpital Saint-Louis, AP-HP, Université Paris Cité	Paris, France	Co-Chair	

Supplemental Online Content: Nonauthor Collaborators
*First name, last name, and suffix (if applicable) are required and will appear in PubMed.

***First Name and Middle Initial(s)**	***Last Name**	***Suffix (eg, Jr, III)**	Academic Degrees	Institution	Location (city, state/province, country)	Role or Contribution, eg, chair, principal investigator	Group (if more than 1 Group listed in the byline) and/or Subgroup (eg, Steering Committee)
Guillaume	Ploussard		M.D., Ph.D.	Department of Urology, La Croix du Sud Hôpital	Quint Fonsegrives, France	Principal investigator	
Andreas	Røder		M.D., Ph.D.	Urological Research Unit, Department of Urology, Copenhagen University Hospital - Rigshospitalet	Copenhagen, Denmark	Principal investigator	
Morgan	Rouprêt		M.D., Ph.D.	Sorbonne University, GRC 5 Predictive Onco-Uro, AP-HP, Urology, Pitie-Salpetriere Hospital, F-75013	Paris, France	Principal investigator	
Maj	Sanson-Stern		M.D.	Department of Urology, University Hospital of Guadeloupe	Pointe-à-Pitre, France	Assistant	
Alberto	Sasia		M.D.	Division of Urology, Department of Surgical Sciences, Molinette Hospital, University of Turin	Torino, Italy	Assistant	
Pietro	Scilipoti		M.D.	Sorbonne University, groupe recherche clinique (GRC) 5 Predictive Onco-Urology, Assistance publique des hôpitaux de Paris (AP-HP), Urology, Pitié-Salpêtrière Hospital	Paris, France	Assistant	
August	Sigle		M.D., M.Sc.	Department of Urology, Faculty of Medicine, University of Freiburg-Medical Centre	Freiburg, Germany	Assistant	
Hein Vincent	Stroomberg		M.D., M.Sc.	Urological Research Unit, Department of Urology, Copenhagen University Hospital - Rigshospitalet	Copenhagen, Denmark	Principal investigator	
Fayek	Taha		M.D., M.Sc.	Department of Urology, Centre Hospitalier Universitaire de Reims	Reims, France	Principal investigator	
Gabrielle	Tissot		M.D., M.Sc.	Department of Urology, Institut Mutualiste Montsouris<br>Department of Urology, Hôpitaux Universitaires Henri Mondor, Assistance Publique - Hôpitaux de Paris, Université Paris-Est Créteil	Paris, France.	Principal investigator	
Julien	Van Damme		M.D., Ph.D.	Cliniques Universitaires Saint-Luc	Brussels, Belgium	Principal investigator	
Thibaut	Waeckel		M.D., Ph.D.	Urology department, CHU de Caen	Caen, France	Principal investigator	

Supplemental Online Content: Nonauthor Collaborators
*First name, last name, and suffix (if applicable) are required and will appear in PubMed.

***First Name and Middle Initial(s)**	***Last Name**	***Suffix (eg, Jr, III)**	Academic Degrees	Institution	Location (city, state/province, country)	Role or Contribution, eg, chair, principal investigator	Group (if more than 1 Group listed in the byline) and/or Subgroup (eg, Steering Committee)
Olivier	Windisch		M.D., Ph.D.	Divison of Urology, Department of Surgery, Geneva University Hospitals, Faculty of Medicine, Geneva University	Geneva, Switzerland	Principal investigator	
