# Supplementary material for: Performance of MRI-Based vs Clinical T Staging in Localized Prostate Cancer
Source: JAMA Netw Open. 2026 Jul 15;9(7):e2623288. doi: 10.1001/jamanetworkopen.2026.23288 (PMC13373662; doi:10.1001/jamanetworkopen.2026.23288)
Supplement: Supplement 3. — Data Sharing Statement [file jamanetwopen-e2623288-s003.pdf]

Data Sharing Statement

Peyrottes. Performance of MRI-Based vs Clinical T Staging in Localized Prostate Cancer. *JAMA Netw Open*. Published July 15, 2026. doi:10.1001/jamanetworkopen.2026.23288

Data
Data available: No

Additional Information
Explanation for why data not available: The data can be made available upon request.
